# Associations of Cognitive Complaints and Depressive Symptoms with Health-Related Quality of Life and Perceived Overall Health in Japanese Adult Volunteers

**DOI:** 10.3390/ijerph18189647

**Published:** 2021-09-13

**Authors:** Kuniyoshi Toyoshima, Takeshi Inoue, Toshiaki Baba, Jiro Masuya, Masahiko Ichiki, Yota Fujimura, Ichiro Kusumi

**Affiliations:** 1 Department of Psychiatry, Hokkaido University Graduate School of Medicine, Sapporo 060-8638, Japan; ikusumi@med.hokudai.ac.jp; 2 Department of Psychiatry, Tokyo Medical University, Tokyo 160-0023, Japan; tinoue@tokyo-med.ac.jp (T.I.); j-masuya@tokyo-med.ac.jp (J.M.); ichiki@tokyo-med.ac.jp (M.I.); fyota@yahoo.co.jp (Y.F.); 3 Bureau of International Health Cooperation, National Center for Global Health and Medicine, Tokyo 162-8655, Japan; baba.toshiaki@gmail.com

**Keywords:** cognitive complaints, depressive symptoms, health-related quality of life, path analysis, mediator

## Abstract

Cognitive complaints, defined as perceived cognitive dysfunction in daily living, are associated with depressive symptoms. The associations of cognitive complaints and depressive symptoms with health-related quality of life (HRQoL) and perceived overall health in Japanese adults remains unknown. To investigate these relationships, we evaluated a convenience sample of 525 Japanese adult volunteers (Mage: 41.3 ± 11.7; 238 male and 287 female). We used the Cognitive Complaints in Bipolar Disorder Rating Assessment (evaluating cognitive complaints), Patient Health Questionnaire-9 (evaluating depressive symptoms), EuroQol-5 Dimension-5 Level (EQ-5D-5L; evaluating HRQoL), and EuroQol-Visual Analogue Scale (EQ-VAS; evaluating perceived overall health). Our path analyses suggested that both cognitive complaints and depressive symptoms had significant total effects on HRQoL and perceived overall health. Furthermore, cognitive complaints were not significantly associated directly with HRQoL and perceived overall health, whereas cognitive complaints were significantly associated with HRQoL and perceived overall health indirectly via depressive symptoms. Depressive symptoms were significantly associated directly with HRQoL and perceived overall health. This study suggests that depressive symptoms may mediate the associations of cognitive complaints with HRQoL and perceived overall health. Thus, to address the HRQoL and perceived overall health associated with cognitive complaints, evaluation and intervention for depressive symptoms may be useful in public health.

## 1. Introduction

Cognitive function has attracted attention in the public health field in recent years [1,2,3,4]. Cognitive impairments have been assessed subjectively and objectively. Subjective cognitive impairments, also known as cognitive complaints, refer to the difficulties a person experiences in completing daily mental tasks [5]. Cognitive complaints have often been considered as measures of cognitive impairments in the general population because they are relatively simple and easy to collect compared with objectively assessed measures of cognitive dysfunction [6]. In Japan, the Cognitive Complaints in Bipolar Disorder Rating Assessment (COBRA) has been used for assessing cognitive complaints in the general adult population [6].

Previous research has indicated a close relationship between cognitive complaints and depressive symptoms. For those with mood disorders, depressive symptoms are correlated with cognitive complaints, and both characteristics are correlated with impaired psychosocial functioning (disability in work, social life, and family life) [7,8]. In the general population, cognitive complaints are highly correlated with depressive symptoms, and both are directly associated with deteriorated psychosocial functioning [6]. A recent study suggested that both depressive symptoms and cognitive complaints directly lead to loss of work productivity among Japanese adult workers [9]. Hence, both cognitive complaints and depressive symptoms are often assessed in public health for individuals with psychiatric illness as well as nonclinical individuals.

Health-related quality of life (HRQoL) has been defined as an individual’s perception of their position in life in the context of the culture and value systems in which they live and in relation to their goals, expectations, standards, and concerns [10]. Unfortunately, HRQoL is considered difficult to improve through medical intervention [11]. Previous studies reported some factors that affect HRQoL [12,13,14,15], and recent studies have emphasized the associations of depressive symptoms and cognitive complaints with HRQoL not only in individuals with mental illness but also in healthy individuals [7,8,16,17]. However, to our knowledge, how depressive symptoms and cognitive complaints are associated with HRQoL in Japanese adults remains relatively unknown.

Health is associated with a wide range of social and economic factors, including income, education, socioeconomic status, retirement, and early life experiences [18]. Physical conditions affect perceived overall health, which is the subjective perception of how healthy, physically and mentally, a person feels [19]. Furthermore, perceived overall health is highly impacted by mental conditions [20]. Previous studies reported that depressive symptoms and cognitive complaints are associated with perceived overall health in older adults [21,22]. However, to our knowledge, how depressive symptoms and cognitive complaints are associated with both HRQoL and perceived overall health in Japanese adults remains relatively unknown.

To schematically summarize the previous studies, the following models were confirmed: “depressive symptoms→HRQoL” [16], “depressive symptoms→perceived overall health” [21], “cognitive complaints→HRQoL” [17], and “cognitive complaints→perceived overall health” [22]. However, the following models were established only among older adults: “cognitive complaints→HRQoL” [17], and “cognitive complaints→perceived overall health” [22]. Furthermore, some models remain to be elucidated among Japanese adults, including: “depressive symptoms→cognitive complaints→HRQoL”, “cognitive complaints→depressive symptoms→HRQoL”, “depressive symptoms→cognitive complaints→perceived overall health”, and “cognitive complaints→depressive symptoms→perceived overall health”. Hence, we aim to assess the mediating roles of depressive symptoms and cognitive complaints using a path analysis to better clarify the associations between cognitive complaints and depressive symptoms with HRQoL and perceived overall health.

## 2. Materials and Methods

### 2.1. Research Participants

Using convenience sampling, we recruited 597 adult volunteers between April 2017 and April 2018 in Tokyo, Japan. Of the 597 participants who agreed to participate, 72 did not complete the study assessments. Our final sample consisted of the 525 participants for whom we had complete clinical and sociodemographic data. This study was part of a larger research endeavor in which several questionnaires were administered [6].

### 2.2. Ethical Considerations

We performed this research at Tokyo Medical University, Tokyo, Japan, with approval from the local ethics committee (approval number: SH3502). All subjects provided written informed consent, and we conducted the research in accordance with the Helsinki Declaration.

### 2.3. Demographic Data

We based the demographic variables included in the analysis on self-reports. Moreover, we divided the annual income into 19 categories from 1 to 19, with 1 indicating an income of less than JPY 500,000, and 19 indicating an income of at least JPY 20,000,000. According to the Federal Reserve historical foreign exchange rates, 1 USD = JPY 106.7754 in 2020 [23]. Social hierarchical awareness comprised 10 social rules wherein participants received 1 point for each answer. Participants were asked, “if you divide the current Japanese society into the following 10 levels, where do you think you fall?”. The scores ranged from 1 to 10, with 10 as the highest score. Nominal variables included sex (1 = male; 2 = female), marital status (0 = single; 1 = married), current employment status (0 = not currently employed; 1 = currently employed), psychiatric history (0 = no; 1 = yes), current psychiatric treatment (0 = not currently receiving treatment; 1 = currently receiving treatment), drinking alcohol (0 = no; 1 = yes), and smoking (0 = no; 1 = yes). Participant demographics are displayed in Table 1.

### 2.4. Assessments

We assessed the participants using the Cognitive Complaints in Bipolar Disorder Rating Assessment (COBRA), a tool for assessing cognitive complaints; the Patient Health Questionnaire-9 (PHQ-9), a tool for assessing depressive symptoms; the EuroQol-5 Dimension-5 Level (EQ-5D-5L), a tool for assessing HRQoL; the EuroQol-Visual Analog Scale (EQ-VAS) for assessing perceived overall health. Cronbach’s α was calculated for the following tools; EQ-5D-5L (0.61), PHQ-9 (0.85), and COBRA score (0.91).

#### 2.4.1. Assessment of Cognitive Complaints

COBRA is a 16-item, self-administered instrument that evaluates cognitive complaints through the performance of daily mental tasks [5]. COBRA item 1 was “Do you have difficulties remembering peoples’ names?”. Participants used a 4-point scale (ranging from 0 = never to 3 = always) to rate each item. The total score was derived by adding the scores of all items. The maximum total score was 48, and a total score of ≥15 was considered an indicator of moderate to severe cognitive dysfunction [24]. In our study, we used the Japanese version of COBRA [25], which was developed and validated for use with the general adult population [6].

#### 2.4.2. Assessment of Depressive Symptoms

PHQ-9 is a self-reported scale that can be utilized to assess depressive symptoms [26]. PHQ-9 item 1 was “little interest or pleasure in doing things”. We used its Japanese version in this study [27] as a screening scale with a cut-off summary score of 10 for depressive symptoms [28]. Similar types of research in the past have also used PHQ-9 for the general population [6].

#### 2.4.3. Assessment of Health-Related Quality of Life

The EuroQol Group developed the EQ-5D [29]. The EQ-5D-5L was developed with five descriptive levels for each health dimension because of the insufficient sensitivity and ceiling effect of the EQ-5D-3L [30,31]. The EQ-5D-5L is a self-administered questionnaire that comprises five dimensions: mobility, self-care, usual activities, pain/discomfort, and anxiety/depression. Each dimension has five levels: no problems, slight problems, moderate problems, severe problems, and extreme problems [30,31]. We calculated the EQ-5D-5L utility scores using Japanese EQ-5D-5L tariffs [31]. The EQ-5D-5L also includes a global health state measurement of perceived overall health based on the EQ-VAS, which has a vertical scale ranging from 0 to 100. A higher EQ-VAS score signifies better health. Respondents marked a point on the EQ-VAS to indicate their perception of their overall health [32]. Regarding the difference between the EQ-5D-5L score and EQ-VAS score, the Japanese preference on quality of life was assessed using the Japanese version of the EQ-5D-5L, which was developed based on Japanese norms. Conversely, the Japanese version of the EQ-VAS provides an estimation of an individual’s general health status, although it was not necessarily reflective of Japanese preference [31,32].

### 2.5. Statistical Analysis

We conducted Pearson’s correlation analysis using Bonferroni correction and forced entry multiple regression analysis to investigate the associations among the depressive symptoms, cognitive complaints, HRQoL, and perceived overall health. In the multiple regression analysis, we confirmed the linearity by performing a normal probability plot. Then, we conducted a path analysis to assess the associations of depressive symptoms and cognitive complaints with HRQoL and perceived overall health. We did not use the goodness-of-fit index because of the saturation models. This study referred to a minimum sample size requirement of 100 because of the saturation model [33,34]. For path analyses, we used the maximum likelihood robust estimation. Lastly, we conducted statistical analyses using Stata MP 16 (Stata Corp LLC, College Station, TX, USA). A *p*-value of <0.05 was deemed statistically significant.

## 3. Results

Clinical and sociodemographic data and cognitive complaints, depressive symptoms, HRQoL, and perceived overall health for the study participants are reported in Table 1.

**Table 1:** Clinical and sociodemographic data (*n* = 525).

Characteristics	Mean (*SD*)	*n* (%)
Age	41.3 (11.7)	
Education (years)	14.7 (1.8)	
Sex, male/female		238 (45.3)/287 (54.7)
Married/not currently married		351 (66.9)/174 (33.1)
Income rank	10.2 (3.9)	
Current employment		516 (98.3)
Social hierarchy	5.1 (1.6)	
Positive psychiatric history		55 (10.5)
Current psychiatric treatment		20 (3.8)
Drinking alcohol		343 (65.3)
Smoking		95 (18.1)
PHQ-9 score	4.0 (4.2)	
EQ-5D-5L score	0.93 (0.09)	
EQ-VAS score	74.9 (14.6)	
COBRA total score	8.4 (6.7)	

*SD*—standard deviation; COBRA—Cognitive Complaints in Bipolar Disorder Rating Assessment; EQ-5D-5L—EuroQol-5 Dimension-5 Level; PHQ-9—Patient Health Questionnaire-9; EQ-VAS—EuroQol Visual Analog Scale.

### 3.1. Associations between Cognitive Complaints, Depressive Symptoms, HRQoL, and Perceived Overall Health

Cognitive complaints were significantly and positively associated with depressive symptoms, whereas they were significantly and negatively associated with HRQoL and perceived overall health. Moreover, depressive symptoms were significantly and negatively associated with HRQoL and perceived overall health. Lastly, HRQoL was significantly and positively associated with perceived overall health (Table 2).

### 3.2. Multiple Regression Analysis of HRQoL and Perceived Overall Health

Depressive symptoms and current employment significantly and negatively predicted HRQoL. Overall health was significantly and negatively predicted by depressive symptoms and was significantly and positively predicted by age, social hierarchy, and drinking alcohol (Table 3).

### 3.3. Path Analysis

We performed path analyses to assess the relationships among cognitive complaints, depressive symptoms, and HRQoL and perceived overall health, in this order.

**Figure 1:**
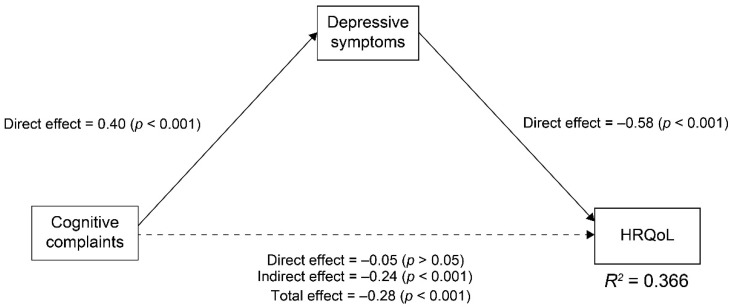
Path analysis of cognitive complaints, depressive symptoms, and HRQoL using COBRA, PHQ-9, and EQ-5D-5L Scores, respectively (*n* = 525). The numbers next to the arrows demonstrate the standardized path coefficients. The solid arrows represent the statistically significant paths and the dashed arrow shows the non-significant path. We did not use the goodness-of-fit index because of the saturation model. *R*^2^ indicates coefficients of determination.

First, we computed the standardized path coefficients using COBRA, PHQ-9, and the EQ-5D-5L scores to investigate the associations between cognitive complaints, depressive symptoms, and HRQoL (Figure 1). In the model, cognitive complaints were significantly associated with depressive symptoms directly (0.40, *p* < 0.001), and depressive symptoms were significantly associated with HRQoL directly (−0.58, *p* < 0.001). Cognitive complaints were significantly associated with HRQoL indirectly via depressive symptoms (−0.24, *p* < 0.001), whereas cognitive complaints were not significantly associated with HRQoL directly (−0.05, *p* > 0.05). The total effect of cognitive complaints on the HRQoL was significant (−0.28, *p* < 0.001). To summarize, cognitive complaints affected HRQoL indirectly via depressive symptoms. In the path analysis, the squared multiple correlation coefficient of HRQoL was 0.366; that is, the model explained 36.6% of the variability in the HRQoL.

**Figure 2:**
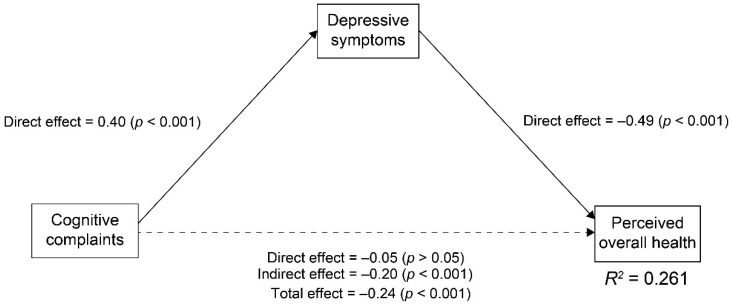
Path Analysis of Cognitive Complaints, Depressive Symptoms, and Perceived Overall Health Using COBRA, PHQ-9, and EQ-VAS Scores, respectively (*n* = 525). The numbers next to the arrows demonstrate the standardized path coefficients. The solid arrows represent the statistically significant paths, and the dashed arrow shows the non-significant path. We did not use the goodness-of-fit index because of the saturation model. *R^2^* indicates coefficients of determination.

Second, we computed the standardized path coefficients using COBRA, PHQ-9, and EQ-VAS scores to investigate the associations of cognitive complaints, depressive symptoms, and perceived overall health (Figure 2). In the model, cognitive complaints significantly influenced depressive symptoms directly (0.40, *p* < 0.001), and depressive symptoms significantly affected overall health directly (−0.49, *p* < 0.001). Cognitive complaints significantly affected overall health indirectly (−0.20, *p* < 0.001), whereas cognitive complaints did not significantly affect overall health directly (−0.05, *p* > 0.05). The total effect of cognitive complaints on overall health was significant (−0.24, *p* < 0.001). To summarize, cognitive complaints affected overall health indirectly via depressive symptoms. In the path analysis, the squared multiple correlation coefficient of overall health was 0.261; that is, the model explained 26.1% of overall health variability.

Third, we performed the path analysis separately for the non-positive psychiatric group (N = 466) and the positive psychiatric group (N = 59). We defined the positive psychiatric group as having a psychiatric history or currently receiving psychiatric treatment. We used the following retrospective questions: “Do you have any mental illness that you have been treated for in the past by going to the hospital or taking prescription medications?” and “Are there any mental illnesses you are currently receiving treatment for by going to the hospital or taking prescription medications?” In the positive psychiatric group, 55 individuals had a psychiatric history and 4 individuals did not have a psychiatric history. Furthermore, 20 individuals were currently receiving psychiatric treatment and 39 individuals were not currently receiving psychiatric treatment. The results of the path analysis are shown in Tables S1–S4. For the non-positive psychiatric group (N = 466), the associations of cognitive complaints and depressive symptoms with health-related quality of life (HRQoL) and perceived overall health were similar to those for our total sample (N = 525) (Tables S1 and S2). For the positive psychiatric group (N = 59), cognitive complaints were significantly and directly associated with HRQoL and perceived overall health (Tables S3 and S4). Hence, the associations of cognitive complaints and depressive symptoms with HRQoL and perceived overall health were different between our total sample (N = 525), non-psychiatric group (N = 466), and psychiatric group (N = 59). However, the non-psychiatric group had a small sample size, which could be a limitation. Hence, the results of the additional path analyses are provided as supplementary materials (Tables S1–S4).

Finally, we performed path analyses to assess the relationships among depressive symptoms, cognitive complaints, and HRQoL and perceived overall health, in this order. We, thus, tested whether cognitive complaints mediated the associations of depressive symptoms with HRQoL and perceived overall health. Table S5 shows the “depressive symptoms→cognitive complaints→HRQoL” model, and Table S6 depicts the “depressive symptoms→cognitive complaints→perceived overall health” model. However, indirect effects from depressive symptoms to HRQoL via cognitive complaints (−0.02, *p* > 0.05) and from depressive symptoms to perceived overall health via cognitive complaints (−0.02, *p* > 0.05) were not statistically significant. Hence, cognitive complaints did not significantly mediate the relation between depressive symptoms and HRQoL and perceived overall health in the models.

## 4. Discussion

We hypothesized that cognitive complaints and depressive symptoms would be directly associated with HRQoL. Our path analyses confirmed that both cognitive complaints and depressive symptoms had significant total effects on HRQoL and perceived overall health. However, our results also suggested that while depressive symptoms are directly associated with HRQoL, cognitive complaints are only indirectly associated with HRQoL via depressive symptoms. Conversely, previous studies have shown that cognitive complaints directly influence work productivity loss and functional disability, and partially mediate the effect of depressive symptoms on work productivity loss in workers [6,9] and functional disability in the general population [6]. Therefore, the relative impact of depressive symptoms and cognitive symptoms may vary; depressive symptoms may have a large impact on HRQoL, whereas cognitive complaints may greatly affect work productivity loss and functional disability. Hence, to address HRQoL associated with cognitive complaints, evaluations and interventions for depressive symptoms may be useful.

We also hypothesized that cognitive complaints and depressive symptoms would be directly associated with perceived overall health. However, such as the findings for HRQoL, our results suggested that depressive symptoms are directly associated with perceived overall health, but cognitive complaints are only indirectly associated with perceived overall health via depressive symptoms. A previous study suggested that both depressive symptoms and cognitive complaints are associated with perceived overall health in healthy older adults and, to a lesser degree, with memory and overall cognitive performance [22]. However, the participants in our study were younger than those in the previous study; that is, participants in this study were over 30 years younger, on average, than those in the previous study [22]. Participants in the previous study also reported fewer cognitive complaints, and more cognitive complaints were associated with depressive symptoms and poor perceived overall health [22]. In the present study, the level of cognitive complaints was relatively mild; hence, cognitive complaints might only be directly associated with depressive symptoms, consistent with the previous study. However, to our knowledge, the finding that depressive symptoms may fully mediate the association of cognitive complaints with perceived overall health in Japanese adult volunteers is new. Thus, to address perceived overall health associated with cognitive complaints, evaluations and interventions for depressive symptoms may be useful.

In summary, our results suggested that depressive symptoms are directly associated with HRQoL and perceived overall health, while cognitive complaints are not directly associated with HRQoL and perceived overall health. Recent research suggested that depressive symptoms are directly associated with subjective well-being and ill-being, and cognitive complaints are directly associated with subjective ill-being but not subjective well-being in Japanese adults [35]. Therefore, subjective well-being and HRQoL or perceived overall health may have similar associations with depressive symptoms and cognitive complaints in Japanese adults. In addition, HRQoL, perceived overall health, and subjective well-being are all important goals in mental health. Recently, cost-effectiveness has been emphasized when considering interventions for HRQoL [12,13,14,15,36,37,38]. Regarding the mental health care system, care managers may play important roles in improving HRQoL and reducing the cost of illness because they are considered to be key health care collaborators in the primary health care system [39]. In the future, larger longitudinal studies are needed to investigate the impact of interventions undertaken by care managers on HRQoL and the cost of illness in Japan.

### Limitations

The heterogeneous characteristics of our sample, which included both healthy and unhealthy individuals, could be a limitation of this study. We used convenience sampling, and 20 individuals (3.8%) were undergoing psychiatric treatment. Therefore, our findings cannot be generalized to nonclinical individuals; that is, those who are not undergoing psychiatric treatment. Moreover, information about pharmacological history, which could play a role as a confounding factor for the statistical analysis, was not collected. The quantitative consumption and the length of time drinking alcohol and smoking were also not collected in this study. For example, we could not control for the presence of medical conditions among teetotalers (“obliged” teetotalers). Lastly, this study was unable to explain the causal linkages among the given parameters because of its cross-sectional design. Thus, longitudinal studies are needed in the future to determine causal relationships.

## 5. Conclusions

This study suggested that both cognitive complaints and depressive symptoms are associated with HRQoL and perceived overall health. Furthermore, depressive symptoms may mediate the associations of cognitive complaints with HRQoL and perceived overall health. To address HRQoL and perceived overall health associated with cognitive complaints, evaluations and interventions for depressive symptoms may be useful.

## Figures and Tables

**Table 2:** Associations between cognitive complaints, depressive symptoms, HRQoL, and perceived overall health (*n* = 525).

	PHQ-9 Score	EQ-5D-5L Score	EQ-VAS Score
COBRA score	0.40 **	−0.28 **	−0.24 **
PHQ-9 score	-	−0.60 **	−0.51 **
EQ-5D-5L score		-	0.53 **
EQ-VAS score			-

** *p* < 0.0001. COBRA—Cognitive Complaints in Bipolar Disorder Rating Assessment; EQ-5D-5L—EuroQol-5 Dimension-5 Level; PHQ-9—Patient Health Questionnaire-9; EQ-VAS—EuroQol Visual Analog Scale. The numerical value indicates Pearson’s *r*.

**Table 3:** Multiple regression analysis of HRQoL and perceived overall health (*n* = 525).

	Dependent Variable: EQ-5D-5L score		Dependent Variable: EQ-VAS score	
	F (13, 511) = 25.6, *p* < 0.0001		F (13, 511) = 16.8, *p* < 0.0001	
**Independent Variables**	***β***	***VIF***	***β***	***VIF***
Age	−0.02	1.47	0.10 *	1.47
Years of education	0.01	1.66	−0.02	1.66
Sex: 1 (Male); 2 (Female)	−0.05	1.27	−0.03	1.27
Married status: 1 (No); 2 (Yes)	0.00	1.35	−0.01	1.35
Income rank	−0.04	1.55	−0.06	1.55
Current employment: 1 (No); 2 (Yes)	−0.10 **	1.04	−0.07	1.04
Social hierarchy	0.05	1.38	0.13 **	1.38
Psychiatric history: 1 (No); 2 (Yes)	0.01	1.32	−0.00	1.32
Current psychiatric treatment: 1 (No); 2 (Yes)	−0.08	1.31	−0.06	1.31
Drinking: 1 (No); 2 (Yes)	0.07	1.21	0.08 *	1.21
Smoking: 1 (No); 2 (Yes)	0.02	1.11	−0.05	1.11
PHQ-9 score	−0.56 **	1.38	−0.44 **	1.38
COBRA score	−0.05	1.24	−0.06	1.24
Adjusted *R^2^*	0.38		0.28	

* *p* < 0.05, ** *p* < 0.01. COBRA—Cognitive Complaints in Bipolar Disorder Rating Assessment; EQ-5D-5L—EuroQol-5 Dimension-5 Level; PHQ-9—Patient Health Questionnaire-9; EQ-VAS—EuroQol Visual Analog Scale; *β*—standard partial regression coefficient; VIF—Variance Inflation Factor.

## Data Availability

The datasets used and/or analyzed during the current study are available from the corresponding author upon reasonable request.

## Supplementary Materials

The following are available online at https://www.mdpi.com/article/10.3390/ijerph18189647/s1, Table S1: standardized path coefficients using COBRA, PHQ-9, and EQ-5D-5L scores (N = 446), Table S2: standardized path coefficients using COBRA, PHQ-9, and EQ-VAS scores (N = 446), Table S3: standardized path coefficients using COBRA, PHQ-9, and EQ-5D-5L scores (N = 59), Table S4: standardized path coefficients using COBRA, PHQ-9, and EQ-VAS scores (N = 59), Table S5: standardized path coefficients using PHQ-9, COBRA, and EQ-5D-5L scores (N = 525), Table S6. standardized path coefficients using PHQ-9, COBRA, and EQ-VAS scores (N = 525).
