# Supplementary material for: Associations of Cognitive Complaints and Depressive Symptoms with Health-Related Quality of Life and Perceived Overall Health in Japanese Adult Volunteers
Source: Int J Environ Res Public Health. 2021 Sep 13;18(18):9647. doi: 10.3390/ijerph18189647 (PMC8468755; doi:10.3390/ijerph18189647)
Supplement: Supplementary file 1 [file ijerph-18-09647-s001.zip › ijerph-1361416-supplementary.pdf]

Table S1. Standardized path coefficients using COBRA, PHQ-9 and EQ-5D-5L scores (N = 446).

	**Direct effect to**	
**From**	PHQ-9	EQ-5D-5L
PHQ-9		−0.57***
COBRA	0.38***	−0.02
	**Indirect effect to (via PHQ-9)**	
**From**		EQ-5D-5L
COBRA		−0.22***
	**Total effect to**	
**From**	PHQ-9	EQ-5D-5L (R^2=0.334)
PHQ-9		−0.57***
COBRA	0.38***	−0.24***

Abbreviations: * $p < 0.05$, ** $p < 0.01$, *** $p < 0.001$.

Table S2. Standardized path coefficients using COBRA, PHQ-9 and EQ-VAS scores (N = 446).

	**Direct effect to**	
**From**	PHQ-9	EQ-VAS
PHQ-9		−0.45***
COBRA	0.38***	−0.02
	**Indirect effect to (via PHQ-9)**	
**From**		EQ-VAS
COBRA		−0.17***
	**Total effect to**	
**From**	PHQ-9	EQ-VAS (R^2=0.210)
PHQ-9		−0.45***
COBRA	0.38***	−0.19***

Abbreviations: * $p < 0.05$, ** $p < 0.01$, *** $p < 0.001$.

Table S3. Standardized path coefficients using COBRA, PHQ-9 and EQ-5D-5L scores (N = 59).

	**Direct effect to**	
**From**	PHQ-9	EQ-5D-5L
PHQ-9		−0.56***
COBRA	0.39**	−0.22*
	**Indirect effect to (via PHQ-9)**	
**From**		EQ-5D-5L
COBRA		−0.22**
	**Total effect to**	
**From**	PHQ-9	EQ-5D-5L (R^2=0.454)
PHQ-9		−0.56***
COBRA	0.39**	−0.44***

Abbreviations: * $p < 0.05$, ** $p < 0.01$, *** $p < 0.001$.

Table S4. Standardized path coefficients using COBRA, PHQ-9 and EQ-VAS scores (N = 59).

	**Direct effect to**	
**From**	PHQ-9	EQ-VAS
PHQ-9		−0.59***
COBRA	0.39**	−0.24*
	**Indirect effect to (via PHQ-9)**	
**From**		EQ-VAS
COBRA		−0.23**
	**Total effect to**	
**From**	PHQ-9	EQ-VAS (R^2=0.524)
PHQ-9		−0.59***
COBRA	0.39**	−0.48***

Abbreviations: * $p < 0.05$, ** $p < 0.01$, *** $p < 0.001$.

Table S5. Standardized path coefficients using PHQ-9, COBRA and EQ-5D-5L scores (N = 525).

	**Direct effect to**	
**From**	COBRA	EQ-5D-5L
COBRA		−0.05
PHQ-9	0.40***	−0.58***
	**Indirect effect to (via COBRA)**	
**From**		EQ-5D-5L
PHQ-9		−0.02
	**Total effect to**	
**From**	COBRA	EQ-5D-5L (R^2=0.366)
COBRA		−0.05
PHQ-9	0.40***	−0.60***

Abbreviations: * $p < 0.05$, ** $p < 0.01$, *** $p < 0.001$.

Table S6. Standardized path coefficients using PHQ-9, COBRA and EQ-VAS scores (N = 525).

	**Direct effect to**	
**From**	COBRA	EQ-VAS
COBRA		−0.05
PHQ-9	0.40***	−0.49***
	**Indirect effect to (via COBRA)**	
**From**		EQ-VAS
PHQ-9		−0.02
	**Total effect to**	
**From**	COBRA	EQ-VAS (R^2=0.261)
COBRA		−0.05
PHQ-9	0.40***	−0.51***

Abbreviations: * $p < 0.05$, ** $p < 0.01$, *** $p < 0.001$.
